# Genome Sequence of Human Rhinovirus A22, Strain Lancaster/2015

**DOI:** 10.1128/genomeA.01713-16

**Published:** 2017-03-23

**Authors:** Kate V. Atkinson, Lisa A. Bishop, Glenn Rhodes, Nicolas Salez, Neil R. McEwan, Matthew J. Hegarty, Julie Robey, Nicola Harding, Simon Wetherell, Robert M. Lauder, Roger W. Pickup, Mark Wilkinson, Derek Gatherer

**Affiliations:** a Division of Biomedical & Life Sciences, Faculty of Health & Medicine, Lancaster University, Lancaster, United Kingdom; b Royal Lancaster Infirmary, Lancaster, United Kingdom; c Centre for Ecology & Hydrology, Lancaster Environment Centre, Lancaster University, Lancaster, United Kingdom; d UMR_D 190, Emergence des Pathologies Virales, Aix-Marseille University, Marseille, France; e Institute of Biological, Environmental & Rural Sciences, Aberystwyth University, Aberystwyth, United Kingdom; f Queen Square Medical Practice, Lancaster, United Kingdom

## Abstract

The genome of human rhinovirus A22 (HRV-A22) was assembled by deep sequencing RNA samples from nasopharyngeal swabs. The assembled genome is 8.7% divergent from the HRV-A22 reference strain over its full length, and it is only the second full-length genome sequence for HRV-A22. The new strain is designated strain HRV-A22/Lancaster/2015.

## GENOME ANNOUNCEMENT

Human rhinovirus A (order *Picornavirales*; family *Picornaviridae*; genus *Enterovirus*; species rhinovirus A) presents a diverse cluster of genotypes (1) and has been implicated in opportunistic infections in immunocompromised patients (2), community-acquired pneumonia of infants (3), chronic respiratory infection in cystic fibrosis (4), and exacerbation of asthma and chronic obstructive pulmonary disease (COPD) (5). It has a positive-sense single-stranded RNA genome of 7.1 kb, encoding a polyprotein that is processed into 11 mature peptides. Rhinovirus A genotype 22 (HRV-A22) has one complete genome sequence in GenBank, that of the ATCC VR-1132 reference strain (accession no. FJ445122). Eleven other fragments have been deposited, ranging in size from 207 to 1,713 bases.

Six volunteers with COPD were recruited from a general practice surgery in Lancaster, United Kingdom (54.05°N, 2.80°W). RNA was obtained from nasopharyngeal swabs taken between 5 January 2015 and 25 February 2015. One patient was asymptomatic at time of sampling, and the other five patients exhibited symptoms consistent with respiratory tract infection. Ethical approval was granted by the UK National Research Ethics Service, reference 14/LO/1634, NIHR Clinical Research Network (UKCRN) Portfolio, ID 17799. All methods were carried out in accordance with the relevant guidelines and regulations.

Pooled RNA underwent deep sequencing using an Illumina Nextera XT library and HiSeq 2500 system (SRA accession no. SRR4733497), and an HRV-A22 genome was assembled using Bowtie 1.1.1 (6) and BWA 0.7.12-r1039 (7), with FJ445122 as the template. The assembled genome is 7,129 bases in length and differs from the reference genome at 620 positions (8.7%), with four gaps. Eleven bases in a run starting at position 2129 could not be resolved. The polyprotein, excluding the unresolved 11 bases, differs from the reference polyprotein at 34 positions (1.6%). The new strain is only the second full-length genome of HRV-A22 and has been designated HRV-A22/Lancaster/2015. Three pediatric patients with cough and other respiratory symptoms were consented to give nasal swabs between 17 December 2014 and 6 February 2015, which were similarly processed (SRA accession no. SRR4733499). Their deep-sequence reads produced partial alignments to HRV-A22/Lancaster/2015 without mismatches. Deep sequencing of seven pooled sets of RNA (SRA accession no. SRP092324) from a further 139 volunteers (of which 63 had respiratory symptoms) yielded no reads aligning to HRV-A22/Lancaster/2015. Finding HRV-A22 in pediatric and COPD deep-sequencing pools only is consistent with the clinical literature on rhinovirus A (3, 5).

McIntyre et al. (8) recommend that a divergence of 10.5% in the VP1 gene should be used as the threshold for designation of a new genotype of human rhinovirus A. We aligned 870 bases of HRV-A22/Lancaster/2015 in VP1 with homologous sequences from HRV-A22 strains KM4 (accession no. KF015733), Ledford et al. (9) (accession no. AY355203), Laine et al. (10) (accession no. AY450473), and ATCC VR-1132 (accession no. FJ445122). Phylogenetic analysis shows that HRV-A22/Lancaster/2015 is a nearest neighbor of strain KM4, (Kunming, China, 2011), at 3.0% divergence, and is 9.7% divergent from the other HRV-A22 strains, in VP1. Lancaster/2015 and KM4 thus represent an outlier group within HRV-A22 but are insufficiently divergent to constitute a novel genotype (BAM files, alignments, and phylogenetic trees are available from https://doi.org/10.17635/lancaster/researchdata/123).

### Accession number(s).

The genome sequence of HRV-A22/Lancaster/2015 has been deposited in GenBank under the accession number KY342346.
